# Statistical Inference of Inverted Exponentiated Rayleigh Distribution under Joint Progressively Type-II Censoring

**DOI:** 10.3390/e24020171

**Published:** 2022-01-24

**Authors:** Jingwen Fan, Wenhao Gui

**Affiliations:** Department of Mathematics, Beijing Jiaotong University, Beijing 100044, China; 19271151@bjtu.edu.cn

**Keywords:** inverted exponentiated Rayleigh distribution, joint progressively type-II censoring scheme, maximum likelihood estimation, Bayesian inference, Bootstrap methods, Monte Carlo simulation

## Abstract

Inverted exponentiated Rayleigh distribution is a widely used and important continuous lifetime distribution, which plays a key role in lifetime research. The joint progressively type-II censoring scheme is an effective method used in the quality evaluation of products from different assembly lines. In this paper, we study the statistical inference of inverted exponentiated Rayleigh distribution based on joint progressively type-II censored data. The likelihood function and maximum likelihood estimates are obtained firstly by adopting Expectation-Maximization algorithm. Then, we calculate the observed information matrix based on the missing value principle. Bootstrap-p and Bootstrap-t methods are applied to get confidence intervals. Bayesian approaches under square loss function and linex loss function are provided respectively to derive the estimates, during which the importance sampling method is introduced. Finally, the Monte Carlo simulation and real data analysis are performed for further study.

## 1. Introduction

### 1.1. Inverted Exponentiated Rayleigh Distribution

Rayleigh distribution is a special form of the Weibull distribution, which was first proposed by Rayleigh when he studied the problems in the field of acoustics. Ref. [1] discussed the generalization of the Rayleigh distribution and its application to practical problems. Ref. [2] introduced Bayesian approaches to study the statistical inference of Rayleigh distribution. Ref. [3] combined progressively type-II censored data with the Rayleigh model and studied the estimations of parameters. Rayleigh distribution has only one parameter, and its applications in practice are limited and not flexible. Many scholars have extended it to two-parameter distributions. Refs. [4,5] began to study the estimations of generalized Rayleigh distribution. Ref. [6] extended progressively type-II censoring scheme to generalized Rayleigh distribution. Recently, under non-informative prior, Ref. [7] studied the estimation of the shape parameter of generalized Rayleigh distribution. Noticing that the hazard function of generalize Rayleigh distribution is monotone when the shape parameter is greater than $\frac{1}{2}$ but the hazard function of inverted exponentiated Rayleigh distribution is nonmonotone, which is more realistic in real life, more scientists become interested in this distribution. Ref. [8] analyzed the estimation of the inverted exponentiated Rayleigh distribution under progressively first-failure censoring scheme and Ref. [9] further studied its prediction. Ref. [10] considered inverted exponentiated Rayleigh distribution with adaptive type-II progressive hybrid censored data. Ref. [11] applied this distribution to analyzing coating weights of iron sheets data.

**Figure 1:**
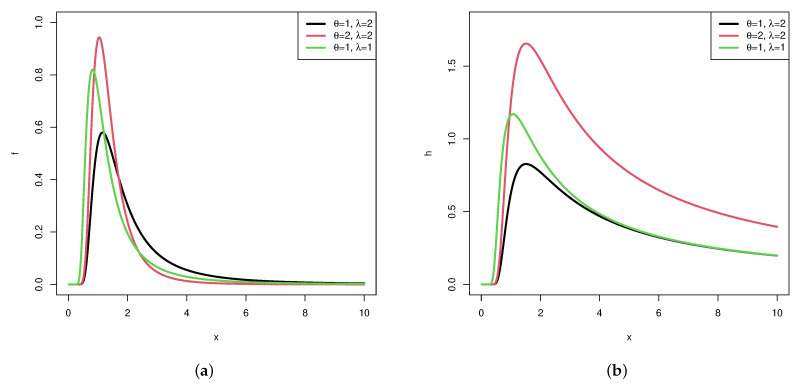
Plots of pdf and hazard function of IERD. (**a**) Pdf of IERD. (**b**) Hazard function of IERD.

A random variable *X* follows inverted exponentiated Rayleigh distribution (IERD) if its probability density function (pdf), the cumulative distribution function (cdf) and hazard function take the forms, respectively, as

(1) $$f\left(x;\theta ,\lambda \right)=2\theta \lambda x^{-3}e^{-\frac{\lambda }{x^{2}}}{\left(1-e^{-\frac{\lambda }{x^{2}}}\right)}^{\theta -1},\;x>0;\;\theta ,\lambda >0,$$

(2) $$F\left(x;\theta ,\lambda \right)=1-{\left(1-e^{-\frac{\lambda }{x^{2}}}\right)}^{\theta },\;x>0;\;\theta ,\lambda >0,$$

(3) $$h\left(x;\theta ,\lambda \right)=2\theta \lambda x^{-3}e^{-\frac{\lambda }{x^{2}}}{\left(1-e^{-\frac{\lambda }{x^{2}}}\right)}^{-1},\;x>0;\;\theta ,\lambda >0,$$

where θ and λ are the shape and scale parameters respectively, and they are both positive. Figure 1 shows the pdfs and hazard functions for different θ and λ of the IERD. It is obvious that both functions are nonmonotone. The IERD can be treated as a useful alternative to some other lifetime distributions such as Weibull distributions, and it has been applied in many fields. Specially, it can effectively be utilized to most experiments of electrical or mechanical devices, patient treatments, and so on.

### 1.2. Joint Progressively Type-II Censoring Scheme

Type-I and type-II censoring schemes have been mostly used. The definition of a type-I censoring scheme is that the observations are terminated at a fixed time and the failure times are recorded. The definition of a type-II censoring scheme is that the observations are terminated until a sufficient and prefixed number of units fail. However, these two censoring schemes do not work well when the lifetimes of tested units are relatively long. More censoring schemes have been proposed later, and the popular and attractive ones are progressive censoring schemes that remove test units every failure time, not just at the last time. Ref. [12] gave details about the progressive censoring schemes. Progressive censoring schemes can be classified as progressively type-I and progressively type-II. The progressively type-I censoring scheme is used in multiple lifetime models, like Refs. [13,14,15]. Many scholars have studied progressively type-II censoring scheme, too. Ref. [16] began to apply it to generalized Gamma distribution. Refs. [17,18] discussed different distributions under progressively type-II censoring scheme.

The censoring schemes mentioned above are all used in one-sample problems. In real life, we face and need to consider two or more samples from different assembly lines. The joint progressively type-II censoring scheme is quite useful in comparing the lifetimes of products from different assembly lines and has received a lot of attention in recent years. Ref. [19] first introduced the joint progressively type-II censoring scheme to study two populations from different exponential distributions. Ref. [20] extended it to multiple exponential populations and provided statistical inference. In the same year, they proposed a new two-sample progressively type-II censoring scheme [21] to study two populations more conveniently. Recently, Ref. [22] discussed the estimation of generalized exponential distribution based on joint progressively type-II censored data. Ref. [23] estimated the two unknown parameters of the inverse exponentiated Rayleigh distribution based on progressive censored data using the pivotal quantity method. In Ref. [24], the reliability of the stress-strength model was introduced and derived for the probability P(T<X<Z) of a component strength *X* relation between two stresses, *T* and *Z*, which follows to have IERD with different unknown shape parameters and common known scale parameter.

**Figure 2:**
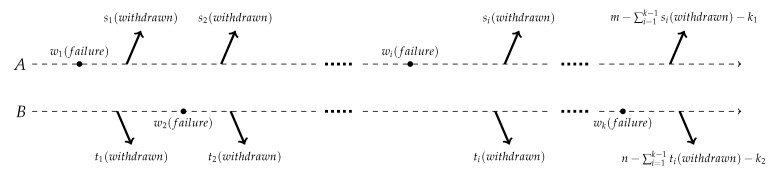
JPC scheme.

The joint progressively type-II censoring (JPC) scheme can be briefly described as follows. Suppose sample A and sample B are taken from two populations. Sample A has *m* units and sample B has *n* units. The two samples are merged and used for a life testing experiment. Set *k* as the amount of failures and let $r_{1},\cdots ,r_{k}$ be the numbers of units withdrawn every time, which satisfy $\sum_{i=1}^{k}\left(r_{i}+1\right)=m+n$. At the time of the first failure $w_{1}$, $r_{1}$ units are intentionally withdrawn from the remaining units. $r_{1}$ units include $s_{1}$ units withdrawn from sample A and $t_{1}$ units withdrawn from sample B. Similarly, at the time of *i*-th failure $w_{i}$ (i=2,3,⋯,k−1), $r_{i}$ units are withdrawn from the remaining $m+n-i-\sum_{l=1}^{i-1}r_{l}$ units. $r_{i}$ units include $s_{i}$ units withdrawn from sample A and $t_{i}$ units withdrawn from sample B. Here $r_{i}$ is prefixed, and $s_{i}$ and $t_{i}$ are random but satisfy $s_{i}+t_{i}$=$r_{i}$. The experiment will be finished at the *k*-th time and we withdraw all the rest of the surviving units. Besides, we let $z_{i}$=1 (or 0) if the *i*-th failure is from sample A (or sample B). According to the scheme above, we introduce three elements to express the observed censored sample, $\left(\left(w_{i},s_{i},z_{i}\right),\cdots ,\left(w_{k},s_{k},z_{k}\right)\right)$. Let $k_{1}=\sum_{i=1}^{k}z_{i}$ be the total number of failures from sample A, and $k_{2}=\sum_{i=1}^{k}\left(1-z_{i}\right)$ be the total number of failures from sample B. Figure 2 below shows the scheme.

In this paper, we make statistical inference and analyze two samples from two-parameter IERD under joint progressively type-II censoring scheme. The maximum likelihood estimation and Bayesian inference are applied to get point estimations and interval estimations. Expectation-Maximization (EM) algorithm is used to calculate the maximum likelihood estimates, which is a three-dimensional optimization problem. Then, the observed information matrix is derived. Bootstrap-p and Bootstrap-t methods are adopted to compute the confidence intervals. In Bootstrap-t method, the observed information matrix obtained is essential. When doing Bayesian inference, non-informative prior and informative prior are provided. With these two Bayesian priors, we obtain Bayesian estimates based on both square loss function and linex loss function. To get the arithmetic solution, importance sampling technique is used. Monte Carlo simulation and real data analysis are performed to compare the performance of different methods.

The rest of the paper is arranged as follows. In Section 2, the EM algorithm is proposed to derive MLEs. In Section 3, we calculate the observed information matrix based on the missing value principle. Then, Bootstrap methods are used to obtain confidence intervals in Section 4. Bayes inference based on non-informative and informative priors is presented in Section 5. In Section 6, the methods above are compared through Monte Carlo simulation and data analysis.

## 2. Maximum Likelihood Estimators and EM Algorithm

Suppose sample A has *m* units and their lifetimes independently follow IERD($\theta_{1},\lambda$) and sample B has *n* units and their lifetimes independently follow IERD($\theta_{2},\lambda$). According to the JPC scheme, we have the observed data $\left(\left(w_{i},s_{i},z_{i}\right),\cdots ,\left(w_{k},s_{k},z_{k}\right)\right)$ for prefixed $r_{1},\cdots ,r_{k}$. Therefore, the likelihood function without the normalizing constant can be written as

$$\begin{array}{c} L\left(\theta_{1},\theta_{2},\lambda \;|data\right)=\prod_{w_{i}\in S}f\left(w_{i};\theta_{1},\lambda \right)\prod_{w_{j}\in T}f\left(w_{j};\theta_{2},\lambda \right)\prod_{l=1}^{k}{\left(1-F(w_{l};\theta_{1},\lambda )\right)}^{s_{l}}{\left(1-F(w_{l};\theta_{2},\lambda )\right)}^{t_{l}}, \end{array}$$

where $s_{l}+t_{l}=r_{l}$ for l=1,⋯,k, *S* means the times of failures from sample A and *T* means the times of failures from sample B. According to (1) and (2), the likelihood function becomes

(4) $$\begin{array}{cc} L(\theta_{1},\theta_{2},\lambda \;|data)= & {\left(2\lambda \right)}^{k}\theta_{1}^{k_{1}}\theta_{2}^{k_{2}}\prod_{i=1}^{k}w_{i}^{-3}e^{-\frac{\lambda }{w_{i}^{2}}}{\left(1-e^{-\frac{\lambda }{w_{i}^{2}}}\right)}^{z_{i}\theta_{1}+\left(1-z_{i}\right)\theta_{2}-1}\\ & \times \prod_{i=1}^{k}{\left(1-e^{-\frac{\lambda }{w_{i}^{2}}}\right)}^{\theta_{1}s_{i}}{\left(1-e^{-\frac{\lambda }{w_{i}^{2}}}\right)}^{\theta_{2}t_{i}}, \end{array}$$

where $k_{1}=\sum_{i=1}^{k}z_{i}$, $k_{2}=\sum_{i=1}^{k}\left(1-z_{i}\right)=k-k_{1}$.

If $k_{1}=0$, $k_{2}=k$, the function becomes the following form:

(5) $$\begin{array}{c} L\left(\theta_{1},\theta_{2},\lambda \;|data\right)={\left(2\theta_{2}\lambda \right)}^{k}\prod_{i=1}^{k}w_{i}^{-3}e^{-\frac{\lambda }{w_{i}^{2}}}{\left(1-e^{-\frac{\lambda }{w_{i}^{2}}}\right)}^{\theta_{2}-1}\prod_{i=1}^{k}{\left(1-e^{-\frac{\lambda }{w_{i}^{2}}}\right)}^{\theta_{1}s_{i}}{\left(1-e^{-\frac{\lambda }{w_{i}^{2}}}\right)}^{\theta_{2}t_{i}}. \end{array}$$

When $s_{i}=0$, ${\left(1-e^{-\frac{\lambda }{w_{i}^{2}}}\right)}^{\theta_{1}s_{i}}=1$. When $s_{i}\neq 0$, ${\left(1-e^{-\frac{\lambda }{w_{i}^{2}}}\right)}^{\theta_{1}s_{i}}$ is a strictly decreasing function of $\theta_{1}$ for a fixed λ and it decreases to 0. So when $k_{1}=0$, $L(\theta_{1},\theta_{2},\lambda \;|data)$ is a strictly decreasing function of $\theta_{1}$ for fixed $\theta_{2}$ and λ, which implies that maximum likelihood estimators (MLEs) do not exist. For $k_{2}=0$, the situation is similar. Thus, we assume that $k_{1}>0,k_{2}>0$ to avoid the trivial cases.

The log-likelihood function can be expressed as

(6) $$\begin{array}{cc} \ln L(\theta_{1},\theta_{2},\lambda \;|data)= & k\ln\left(2\lambda \right)+k_{1}\ln\theta_{1}+k_{2}\ln\theta_{2}\\ \; & +\sum_{i=1}^{k}\left[-3\ln w_{i}-\frac{\lambda }{w_{i}^{2}}+\left(z_{i}\theta_{1}+\left(1-z_{i}\right)\theta_{2}-1\right)\ln\left(1-e^{-\frac{\lambda }{w_{i}^{2}}}\right)\right]\\ \; & +\sum_{i=1}^{k}\left[\theta_{1}s_{i}\ln\left(1-e^{-\frac{\lambda }{w_{i}^{2}}}\right)+\theta_{2}t_{i}\ln\left(1-e^{-\frac{\lambda }{w_{i}^{2}}}\right)\right]. \end{array}$$

Take the partial derivatives of $\ln L(\theta_{1},\theta_{2},\lambda \;|data)$, let them equal 0 and the roots of the equations are the maximum likelihood estimates of $\left(\theta_{1},\theta_{2},\lambda \right)$. It is found that the equations are non-linear, and it is infeasible to calculate the solutions directly. The New-Raphson method needs second partial derivatives to inevitably calculate and the computation is cumbersome.

### EM Algorithm

In this subsection, we use the EM algorithm to get MLEs. Ref. [25] proposed the method to get maximum likelihood from incomplete data and Ref. [26] introduced its applications in a generalized partial credit model. The observed data are available and the missing data are the lifetimes of those units withdrawn. Complete data of the experiment consist of the observed data $\left(\left(w_{1},s_{1},z_{1}\right),\cdots ,\left(w_{k},s_{k},z_{k}\right)\right)$ and missing data. At the time of *i*-th failure, $s_{i}$ units are withdrawn from sample A (i=1,2,⋯,k). Assume that the lifetimes of the $s_{i}$ units are $\left(u_{i1},u_{i2},\cdots ,u_{is_{i}}\right)$. Similarly, $t_{i}$ units are withdrawn from sample B. Assume that the lifetimes of the $t_{i}$ units are $\left(v_{i1},v_{i2},\cdots ,v_{it_{i}}\right)$. The missing data are $(\left(u_{11},u_{12},\cdots ,u_{1s_{1}}\right),\cdots ,$$\left(u_{k1},u_{k2},\cdots ,u_{ks_{k}}\right),\left(v_{11},v_{12},\cdots ,v_{1t_{1}}\right),\cdots ,\left(v_{k1},v_{k2},\cdots ,v_{kt_{k}}\right))$ which can be expressed as $\left(U_{1},\cdots ,U_{k},V_{1},\cdots ,V_{k}\right)=\left(U,V\right)$. The complete data $data^{*}$(say) are $(\left(w_{1},s_{1},z_{1}\right),\cdots ,$$\left(w_{k},s_{k},z_{k}),U,V\right)$. The log-likelihood function for the complete data is obtained as

(7) $$\begin{array}{cc} \ln L_{c}\left(\theta_{1},\theta_{2},\lambda \;|data^{*}\right)= & \left(m+n\right)\ln\left(2\lambda \right)+m\ln\theta_{1}+n\ln\theta_{2}-3\sum_{i=1}^{k}\left(\sum_{j=1}^{s_{i}}\ln u_{ij}+\sum_{l=1}^{t_{i}}\ln v_{il}+\ln w_{i}\right)\\ \; & -\lambda \sum_{i=1}^{k}\left(\sum_{j=1}^{s_{i}}\frac{1}{u_{ij}^{2}}+\sum_{l=1}^{t_{i}}\frac{1}{v_{il}^{2}}+\frac{1}{w_{i}^{2}}\right)+\left(\theta_{1}-1\right)\sum_{i=1}^{k}\sum_{j=1}^{s_{i}}\ln\left(1-e^{\frac{-\lambda }{u_{ij}^{2}}}\right)\\ \; & +\left(\theta_{2}-1\right)\sum_{i=1}^{k}\sum_{l=1}^{t_{i}}\ln\left(1-e^{\frac{-\lambda }{v_{il}^{2}}}\right)+\sum_{i=1}^{k}\left(z_{i}\theta_{1}+\left(1-z_{i}\right)\theta_{2}-1\right)\ln\left(1-e^{\frac{-\lambda }{w_{i}^{2}}}\right). \end{array}$$

The EM algorithm is divided into two steps. The pseudo log-likelihood function at the ‘E’-step is given by:

(8) $$\begin{array}{cc} l_{c}\left(\theta_{1},\theta_{2},\lambda |data^{*}\right)= & \left(m+n\right)\ln\left(2\lambda \right)+m\ln\theta_{1}+n\ln\theta_{2}-3\sum_{i=1}^{k}\left[s_{i}E\left(\ln U_{i}|U_{i}>w_{i}\right)+t_{i}E\left(\ln V_{i}|V_{i}>w_{i}\right)\right]\\ \; & -3\sum_{i=1}^{k}\ln w_{i}-\lambda \sum_{i=1}^{k}\left[s_{i}E\left(\frac{1}{u_{ij}^{2}}\right)+t_{i}E\left(\frac{1}{V_{i}^{2}}|V_{i}>w_{i}\right)+\frac{1}{w_{i}^{2}}\right]\\ \; & +\left(\theta_{1}-1\right)\sum_{i=1}^{k}s_{i}E\left(\ln\left(1-e^{\frac{-\lambda }{U_{i}^{2}}}\right)|U_{i}>w_{i}\right)+\left(\theta_{2}-1\right)\sum_{i=1}^{k}t_{i}E\left(\ln\left(1-e^{\frac{-\lambda }{V_{i}^{2}}}\right)|V_{i}>w_{i}\right)\\ \; & +\sum_{i=1}^{k}\left(z_{i}\theta_{1}+\left(1-z_{i}\right)\theta_{2}-1\right)\ln\left(1-e^{\frac{-\lambda }{w_{i}^{2}}}\right). \end{array}$$

The conditional pdfs of the $U_{ij}$ and $V_{il}$ are expressed respectively as (see [27])

$$\begin{array}{c} f_{U_{ij}|\left(W_{1},S_{1},Z_{1}\right),\cdots ,\left(W_{k},S_{k},Z_{k}\right)}\left(u_{ij}|\left(w_{1},s_{1},z_{1}\right),\cdots ,\left(w_{k},s_{k},z_{k}\right)\right)=f_{U_{ij}|W_{i}}\left(u_{ij}|w_{i}\right)=\frac{f_{IERD}\left(u_{ij};\theta_{1},\lambda \right)}{1-F_{IERD}\left(w_{i};\theta_{1},\lambda \right)}, \end{array}$$

$$\begin{array}{c} f_{V_{il}|\left(W_{1},S_{1},Z_{1}\right),\cdots ,\left(W_{k},S_{k},Z_{k}\right)}\left(v_{il}|\left(w_{1},s_{1},z_{1}\right),\cdots ,\left(w_{k},s_{k},z_{k}\right)\right)=f_{V_{il}|W_{i}}\left(v_{il}|w_{i}\right)=\frac{f_{IERD}\left(v_{il};\theta_{2},\lambda \right)}{1-F_{IERD}\left(w_{i};\theta_{2},\lambda \right)}, \end{array}$$

for i=1,⋯,k.

Then, the following formulas are obtained:

$$\begin{array}{c} E\left(\ln U_{ij}|U_{ij}>w_{i}\right)=\int_{w_{i}}^{\infty }\frac{2\theta_{1}\lambda u_{ij}^{-3}\ln u_{ij}e^{\frac{-\lambda }{u_{ij}^{2}}}{\left(1-e^{\frac{-\lambda }{u_{ij}^{2}}}\right)}^{\theta_{1}-1}}{{\left(1-e^{\frac{-\lambda }{w_{i}^{2}}}\right)}^{\theta_{1}}}du_{ij}, \end{array}$$

$$\begin{array}{c} E\left(\frac{1}{U_{ij}^{2}}|U_{ij}>w_{i}\right)=\int_{w_{i}}^{\infty }\frac{2\theta_{1}\lambda u_{ij}^{-5}e^{\frac{-\lambda }{u_{ij}^{2}}}{\left(1-e^{\frac{-\lambda }{u_{ij}^{2}}}\right)}^{\theta_{1}-1}}{{\left(1-e^{\frac{-\lambda }{w_{i}^{2}}}\right)}^{\theta_{1}}}du_{ij}, \end{array}$$

$$\begin{array}{c} E\left(\ln\left(1-e^{\frac{-\lambda }{U_{ij}^{2}}}\right)|U_{ij}>w_{i}\right)=\int_{w_{i}}^{\infty }\frac{2\theta_{1}\lambda u_{ij}^{-3}\ln\left(1-e^{\frac{-\lambda }{u_{ij}^{2}}}\right)e^{\frac{-\lambda }{u_{ij}^{2}}}{\left(1-e^{\frac{-\lambda }{u_{ij}^{2}}}\right)}^{\theta_{1}-1}}{{\left(1-e^{\frac{-\lambda }{w_{i}^{2}}}\right)}^{\theta_{1}}}du_{ij}. \end{array}$$

The expectations related to the functions of $V_{il}$ are similar and omitted here.

In the ‘M’ step, we calculate the estimates of $\theta_{1}$, $\theta_{2}$ and λ, which maximize the pseudo log-likelihood function with finite iterations. Take partial derivatives of (8) to get the expressions of $\theta_{1}$ and $\theta_{2}$ in terms of λ:

(9) $$\begin{array}{c} \hat{\theta_{1}}\left(\lambda \right)=\theta_{1}=\frac{-m}{\sum_{i=1}^{k}s_{i}E\left(\ln\left(1-e^{\frac{-\lambda }{U_{i}^{2}}}\right)|U_{i}>w_{i}\right)+\sum_{i=1}^{k}z_{i}\ln\left(1-e^{\frac{-\lambda }{w_{i}^{2}}}\right)}, \end{array}$$

(10) $$\begin{array}{c} \hat{\theta_{2}}\left(\lambda \right)=\theta_{2}=\frac{-n}{\sum_{i=1}^{k}t_{i}E\left(\ln\left(1-e^{\frac{-\lambda }{V_{i}^{2}}}\right)|V_{i}>w_{i}\right)+\sum_{i=1}^{k}\left(1-z_{i}\right)\ln\left(1-e^{\frac{-\lambda }{w_{i}^{2}}}\right)}. \end{array}$$

Equation (8) can be rewritten as the function only for λ and the problem turns to be a one-dimensional optimization problem. At the *q*-th iteration (q=1,2,⋯), let $\left(\theta_{1}^{\left(q\right)},\theta_{2}^{\left(q\right)},\lambda^{\left(q\right)}\right)$ be the estimates of $\left(\theta_{1},\theta_{2},\lambda \right)$. Plug $\left(\theta_{1}^{\left(q-1\right)},\theta_{2}^{\left(q-1\right)}\right)$ into (8), maximize the function and obtain $\lambda^{\left(q\right)}$. For fixed $\theta_{1}^{\left(q-1\right)}$, $\theta_{2}^{\left(q-1\right)}$ and $\lambda^{\left(q\right)}$, $\theta_{1}^{\left(q\right)}$ and $\theta_{2}^{\left(q\right)}$ can be derived by using the formulas below:

(11) $$\begin{array}{c} \theta_{1}^{\left(q\right)}=\frac{-m}{\sum_{i=1}^{k}s_{i}E_{\left(\theta_{1}^{\left(q-1\right)},\theta_{2}^{\left(q-1\right)},\lambda^{\left(q\right)}\right)}\left(\ln\left(1-e^{\frac{-\lambda }{U_{i}^{2}}}\right)|U_{i}>w_{i}\right)+\sum_{i=1}^{k}z_{i}\ln\left(1-e^{\frac{-\lambda^{\left(q\right)}}{w_{i}^{2}}}\right)}, \end{array}$$

(12) $$\begin{array}{c} \theta_{2}^{\left(q\right)}=\frac{-n}{\sum_{i=1}^{k}t_{i}E_{\left(\theta_{1}^{\left(q-1\right)},\theta_{2}^{\left(q-1\right)},\lambda^{\left(q\right)}\right)}\left(\ln\left(1-e^{\frac{-\lambda }{V_{i}^{2}}}\right)|V_{i}>w_{i}\right)+\sum_{i=1}^{k}\left(1-z_{i}\right)\ln\left(1-e^{\frac{-\lambda^{\left(q\right)}}{w_{i}^{2}}}\right)}. \end{array}$$

The iterations are stopped until $|\lambda^{\left(p\right)}-\lambda^{\left(p-1\right)}|⩽0.0001$, $|\theta_{1}^{\left(p\right)}-\theta_{1}^{\left(p-1\right)}|⩽0.0001$, $|\theta_{2}^{\left(p\right)}-\theta_{2}^{\left(p-1\right)}|⩽0.0001$. $\theta_{1}^{\left(p\right)},\theta_{2}^{\left(p\right)},\lambda^{\left(p\right)}$ are the maximum likelihood estimates of $\theta_{1},\theta_{2},\lambda$.

## 3. Observed Fisher Information Matrix

In this section, the observed information matrix is calculated and will be used in Section 4. According to the idea of [28], we have

$$\begin{array}{c} I_{o}\left(\theta_{1},\theta_{2},\lambda \right)=mI_{1}\left(\theta_{1},\theta_{2},\lambda \right)+nI_{2}\left(\theta_{1},\theta_{2},\lambda \right)-\left(\sum_{i=1}^{k}\sum_{j=1}^{s_{i}}I_{u_{ij}|w_{i}}\left(\theta_{1},\theta_{2},\lambda \right)+\sum_{i=1}^{k}\sum_{l=1}^{t_{i}}I_{v_{il}|w_{i}}\left(\theta_{1},\theta_{2},\lambda \right)\right), \end{array}$$

and the notations below:

$I_{c}$: the observed information matrix of complete data;

$I_{1}$: the observed information matrix of one unit from sample A;

$I_{2}$: the observed information matrix of one unit from sample B;

$I_{o}$: the observed information matrix of observed data;

$I_{m}$: the observed information matrix of missing data.

For sample A,

$$\begin{array}{c} I_{1}=\left[\begin{array}{ccc} I_{11}^{\left(1\right)} & 0 & I_{13}^{\left(1\right)}\\ 0 & 0 & 0\\ I_{31}^{\left(1\right)} & 0 & I_{33}^{\left(1\right)} \end{array}\right] \end{array}$$

where

$I_{11}^{\left(1\right)}=-E\left(\frac{\partial^{2}\ln f_{IERD}\left(x;\theta_{1},\lambda \right)}{\partial \theta_{1}^{2}}\right),I_{13}^{\left(1\right)}=I_{31}^{\left(1\right)}=-E\left(\frac{\partial^{2}\ln f_{IERD}\left(x;\theta_{1},\lambda \right)}{\partial \theta_{1}\partial \lambda }\right),I_{33}^{\left(1\right)}=$$-E(\frac{\partial^{2}\ln f_{IERD}\left(x;\theta_{1},\lambda \right)}{\partial \lambda^{2}})$.  

The missing observed matrices can be obtained as follow:

$$\begin{array}{c} I_{u_{ij}|w_{i}}\left(\theta_{1},\theta_{2},\lambda \right)=\left[\begin{array}{ccc} I_{11}^{u} & 0 & I_{13}^{u}\\ 0 & 0 & 0\\ I_{31}^{u} & 0 & I_{33}^{u} \end{array}\right] \end{array}$$

where

$I_{11}^{u}=-E\left(\frac{\partial^{2}\ln f_{CPF}\left(x;\theta_{1},\lambda \right)}{\partial \theta_{1}^{2}}\right),I_{13}^{u}=I_{31}^{u}=-E\left(\frac{\partial^{2}\ln f_{CPF}\left(x;\theta_{1},\lambda \right)}{\partial \theta_{1}\partial \lambda }\right),I_{33}^{u}=-E\left(\frac{\partial^{2}\ln f_{CPF}\left(x;\theta_{1},\lambda \right)}{\partial \lambda^{2}}\right)$.

Here, CPF means the conditional pdf. Expressions of all the expectations related to $\theta_{1}$ are given in Appendix A. The expressions above related to sample B are similar and omitted.

After getting the observed information matrix, for every fixed $\left(\theta_{1},\theta_{2},\lambda \right)$, the covariance matrix of estimators is the inverse matrix of the observed information matrix.

## 4. Bootstrap Methods

In this section, the Bootstrap methods are introduced to construct confidence intervals. The algorithms of Bootstrap-p method and Bootstrap-t method are respectively given in Algorithms 1 and 2.

- **Bootstrap-p method:**
**Algorithm 1** The algorithm of Bootstrap-p method.
Step 1: Generate two random samples, which are from IERD($\theta_{1},\lambda$) and IERD($\theta_{2},\lambda$), respectively, and use the JPC scheme to get the observed data.
Step 2: Calculate the MLEs (say $\left(\hat{\theta_{1}},\hat{\theta_{2}},\hat{\lambda }\right)$).
Step 3: Use $\left(\hat{\theta_{1}},\hat{\theta_{2}},\hat{\lambda }\right)$ to generate two new samples, respectively.
Step 4: Get new MLEs $\left({\hat{\theta_{1}}}^{\left(i\right)},{\hat{\theta_{2}}}^{\left(i\right)},{\hat{\lambda }}^{\left(i\right)}\right)$.
Step 5: Repeat steps 3 and 4 *N* times.
Step 6: Get the results $\left(\left({\hat{\theta_{1}}}^{\left(1\right)},{\hat{\theta_{2}}}^{\left(1\right)},{\hat{\lambda }}^{\left(1\right)}\right),\cdots ,\left({\hat{\theta_{1}}}^{\left(N\right)},{\hat{\theta_{2}}}^{\left(N\right)},{\hat{\lambda }}^{\left(N\right)}\right)\right)$.
Step 7: Sort $\left({\hat{\theta_{1}}}^{\left(1\right)},\cdots ,{\hat{\theta_{1}}}^{\left(N\right)}\right),\left({\hat{\theta_{2}}}^{\left(1\right)},\cdots ,{\hat{\theta_{2}}}^{\left(N\right)}\right),\left({\hat{\lambda }}^{\left(1\right)},\cdots ,{\hat{\lambda }}^{\left(N\right)}\right)$ in ascending order as
$\left({\hat{\theta_{1}}}_{\left(1\right)},\cdots ,{\hat{\theta_{1}}}_{\left(N\right)}\right),\left({\hat{\theta_{2}}}_{\left(1\right)},\cdots ,{\hat{\theta_{2}}}_{\left(N\right)}\right),\left({\hat{\lambda }}_{\left(1\right)},\cdots ,{\hat{\lambda }}_{\left(N\right)}\right)$.
Step 8: The 100(1−α)% symmetric Bootstrap-p confidence intervals for $\left(\theta_{1},\theta_{2},\lambda \right)$ are
(13)$$\begin{array}{c} \left[{\hat{\theta_{1}}}_{\left(lb\right)},{\hat{\theta_{1}}}_{\left(hb\right)}\right]\;,\;\left[{\hat{\theta_{2}}}_{\left(lb\right)},{\hat{\theta_{2}}}_{\left(hb\right)}\right]\;and\;\left[{\hat{\lambda }}_{\left(lb\right)},{\hat{\lambda }}_{\left(hb\right)}\right], \end{array}$$
where $lb=\left[\frac{\alpha }{2}N\right]$, $hb=\left[\frac{1-\alpha }{2}N\right]$. Here $\left[x\right]$ means the largest integer not exceeding *x*.

- **Bootstrap-t method:**
**Algorithm 2** The algorithm of Bootstrap-t method.
Steps 1 to 5 are the same as those in Algorithm 1.
Step 6: Get the results $\left(\left({\hat{\theta_{1}}}^{\left(1\right)},{\hat{\theta_{2}}}^{\left(1\right)},{\hat{\lambda }}^{\left(1\right)}\right),\cdots ,\left({\hat{\theta_{1}}}^{\left(N\right)},{\hat{\theta_{2}}}^{\left(N\right)},{\hat{\lambda }}^{\left(N\right)}\right)\right)$ and
$\left(Cov^{\left(1\right)},Cov^{\left(2\right)},\cdots ,Cov^{\left(N\right)}\right)$, where $Cov^{\left(i\right)}$ is given by
(14)$$\begin{array}{c} Cov^{\left(i\right)}=I_{o}^{-1}\left({\hat{\theta_{1}}}^{\left(i\right)},{\hat{\theta_{2}}}^{\left(i\right)},{\hat{\lambda }}^{\left(i\right)}\right) \end{array}$$
where $I_{o}$ is obtained in Section 3.
Step 7: $Var({\hat{\theta_{1}}}^{\left(i\right)})$, $Var({\hat{\theta_{2}}}^{\left(i\right)})$ and $Var({\hat{\lambda }}^{\left(i\right)})$ which are diagonal elements of $Cov^{\left(i\right)}$ can be obtained. Define
$$\begin{array}{c} T_{j}^{\left(i\right)}=\frac{{\hat{\theta_{j}}}^{\left(i\right)}-\hat{\theta_{j}}}{\sqrt{Var({\hat{\theta_{1}}}^{\left(i\right)})}},\;for\;j=1,2 \end{array}$$
and
$$\begin{array}{c} T_{\lambda }^{\left(i\right)}=\frac{{\hat{\lambda }}^{\left(i\right)}-\hat{\lambda }}{\sqrt{Var({\hat{\lambda }}^{\left(i\right)})}}. \end{array}$$
Step 8: Sort $\left(T_{1}^{\left(1\right)},\cdots ,T_{1}^{\left(N\right)}\right),\left(T_{2}^{\left(1\right)},\cdots ,T_{2}^{\left(N\right)}\right),\left(T_{\lambda }^{\left(1\right)},\cdots ,T_{\lambda }^{\left(N\right)}\right)$ in ascending order as
$\left(T_{1(1)},\cdots ,T_{1(N)}\right),\left(T_{2(1)},\cdots ,T_{2(N)}\right),\left(T_{\lambda (1)},\cdots ,T_{\lambda (N)}\right)$.
Step 9: The 100(1−α)% Bootstrap-t confidence intervals for $\left(\theta_{1},\theta_{2},\lambda \right)$ are given by
(15)$$\begin{array}{c} \left[\hat{\theta_{j}}-\sqrt{Var(\hat{\theta_{j}})}T_{j(hb)},\hat{\theta_{j}}-\sqrt{Var(\hat{\theta_{j}})}T_{j(lb)}\right]\;for\;j=1,2, \end{array}$$
and
(16)$$\begin{array}{c} \left[\hat{\lambda }-\sqrt{Var(\hat{\lambda })}T_{\lambda (hb)},\hat{\lambda }-\sqrt{Var(\hat{\lambda })}T_{\lambda (lb)}\right]. \end{array}$$
where $lb=\left[\frac{\alpha }{2}N\right]$, $hb=\left[\frac{1-\alpha }{2}N\right]$. Here $\left[x\right]$ means the largest integer not exceeding *x*.

## 5. Bayesian Inference

In this section, we study the Bayesian inference for the three parameters based on non-informative prior and informative prior. The estimates under symmetric loss function (square loss function) and asymmetric loss function (linex loss function) are derived.

Notice that all the parameters range from 0 to +∞, so let Gamma distributions be the prior distributions. Gamma distribution (Ga(α,β)) has the following density:

(17) $$\begin{array}{c} f\left(x|\alpha ,\beta \right)=\frac{\beta^{\alpha }x^{\alpha -1}e^{-\beta x}}{\Gamma (\alpha )},\;x⩾0,\alpha >0,\beta >0, \end{array}$$

It is assumed that $\theta_{1}$, $\theta_{2}$ and λ are independent and follow different Gamma distributions. $\theta_{1}∼Ga\left(a_{1},b_{1}\right)$, $\theta_{2}∼Ga\left(a_{2},b_{2}\right)$ and λ∼Ga(c,d). The hyperparameters $a_{1},b_{1},a_{2},b_{2},c,d>0$. The priors considered are conjugate.

The joint posterior density is given by

(18) $$\begin{array}{cc} \pi (\theta_{1},\theta_{2},\lambda |data)\propto & \pi \left(\theta_{1},\theta_{2},\lambda \right)L\left(\theta_{1},\theta_{2},\lambda |data\right)\\ \propto & \theta_{1}^{a_{1}+k_{1}-1}e^{-\left(b_{1}-\sum_{i=1}^{k}\left(z_{i}+s_{i}\right)\ln\left(1-e^{-\frac{\lambda }{w_{i}^{2}}}\right)\right)\theta_{1}}\times \theta_{2}^{a_{2}+k_{2}-1}e^{-\left(b_{2}-\sum_{i=1}^{k}\left(1-z_{i}+t_{i}\right)\ln\left(1-e^{-\frac{\lambda }{w_{i}^{2}}}\right)\right)\theta_{2}}\\ \; & \times \lambda^{k+c-1}e^{-(d+\sum_{i=1}^{k}\frac{1}{w_{i}^{2}})\lambda }\times \frac{1}{\prod_{i=1}^{k}\left(1-e^{-\frac{\lambda }{w_{i}^{2}}}\right)}\\ = & \pi \left(\theta_{1}|\lambda ,data\right)\times \pi \left(\theta_{2}|\lambda ,data\right)\times \pi \left(\lambda |data\right), \end{array}$$

where $\pi (\theta_{1},\theta_{2},\lambda )$ means the joint prior density.

Observing that in (18) the first two items are Gamma densities, in fact, we check the shape and scale parameters and confirm that $a_{1}+k_{1}>0$, $a_{2}+k_{2}>0$, $b_{1}-\sum_{i=1}^{k}\left(z_{i}+s_{i}\right)\ln\left(1-e^{-\frac{\lambda }{w_{i}^{2}}}\right)>0$ and $b_{2}-\sum_{i=1}^{k}\left(1-z_{i}+t_{i}\right)\ln\left(1-e^{-\frac{\lambda }{w_{i}^{2}}}\right)>0$.

Some facts are obtained:

(19) $$\begin{array}{c} \pi \left(\theta_{1}|\lambda ,data\right)=Ga\left(a_{1}+k_{1},b_{1}-\sum_{i=1}^{k}\left(s_{i}+z_{i}\right)\ln\left(1-e^{-\frac{\lambda }{w_{i}^{2}}}\right)\right), \end{array}$$

(20) $$\begin{array}{c} \pi \left(\theta_{2}|\lambda ,data\right)=Ga\left(a_{2}+k_{2},b_{2}-\sum_{i=1}^{k}\left(t_{i}+1-z_{i}\right)\ln\left(1-e^{-\frac{\lambda }{w_{i}^{2}}}\right)\right), \end{array}$$

(21) $$\begin{array}{cc} \pi (\lambda |data)\propto & \frac{\lambda^{c+k-1}e^{-(d+\sum_{i=1}^{k}\frac{1}{w_{i}^{2}})\lambda }}{\prod_{i=1}^{k}\left(1-e^{-\frac{\lambda }{w_{i}^{2}}}\right)}\times {\left[b_{1}-\sum_{i=1}^{k}\left(s_{i}+z_{i}\right)\ln\left(1-e^{-\frac{\lambda }{w_{i}^{2}}}\right)\right]}^{-(a_{1}+k_{1})}\\ \; & \times {\left[b_{2}-\sum_{i=1}^{k}\left(t_{i}+1-z_{i}\right)\ln\left(1-e^{-\frac{\lambda }{w_{i}^{2}}}\right)\right]}^{-(a_{2}+k_{2})}, \end{array}$$

The distributions of $\theta_{1}$ and $\theta_{2}$ both depend on λ. Generating the random variates of λ is the key. The well-known log-concave method is considered and discussed first.

$\ln\left(\pi (\lambda |data)\right)$ can be written as

$$\begin{array}{cc} \ln\left(\pi (\lambda |data)\right)= & \ln C+\left(k+c-1\right)\ln\lambda -\left(d+\sum_{i=1}^{k}\frac{1}{w_{i}^{2}}\right)\lambda -\sum_{i=1}^{k}\ln\left(1-e^{-\frac{\lambda }{w_{i}^{2}}}\right)\\ \; & -\left(a_{1}+k_{1}\right)\ln\left(b_{1}-\sum_{i=1}^{k}\left(s_{i}+z_{i}\right)\ln\left(1-e^{-\frac{\lambda }{w_{i}^{2}}}\right)\right)\\ \; & -\left(a_{2}+k_{2}\right)\ln\left(b_{2}-\sum_{i=1}^{k}\left(t_{i}+1-z_{i}\right)\ln\left(1-e^{-\frac{\lambda }{w_{i}^{2}}}\right)\right), \end{array}$$

where *C* means the normalization constant of π(λ|data) and the second-order partial derivative of $\ln\left(\pi (\lambda |data)\right)$ is given by

(22) $$\begin{array}{cc} \frac{\partial^{2}\ln\left(\pi (\lambda |data)\right)}{\partial \lambda^{2}}=\; & \sum_{i=1}^{k}\frac{e^{-\frac{\lambda }{w_{i}^{2}}}}{w_{i}^{4}{\left(1-e^{-\frac{\lambda }{w_{i}^{2}}}\right)}^{2}}+\frac{k+c-1}{\lambda^{2}}+\left(a_{1}+k_{1}\right)\left\{{\left[\sum_{i=1}^{k}\left(s_{i}+z_{i}\right)\frac{e^{-\frac{\lambda }{w_{i}^{2}}}}{w_{i}^{2}\left(1-e^{-\frac{\lambda }{w_{i}^{2}}}\right)}\right]}^{2}\right.\\ \; & \left.-\sum_{i=1}^{k}\left[\left(s_{i}+z_{i}\right)\frac{e^{-\frac{\lambda }{w_{i}^{2}}}}{w_{i}^{4}{\left(1-e^{-\frac{\lambda }{w_{i}^{2}}}\right)}^{2}}\times \left(b_{1}-\sum_{i=1}^{k}\left(s_{i}+z_{i}\right)\ln\left(1-e^{-\frac{\lambda }{w_{i}^{2}}}\right)\right)\right]\right\}\\ \; & \times {\left[b_{1}-\sum_{i=1}^{k}\left(s_{i}+z_{i}\right)\ln\left(1-e^{-\frac{\lambda }{w_{i}^{2}}}\right)\right]}^{-2}+\left(a_{2}+k_{2}\right)\left\{{\left[\sum_{i=1}^{k}\left(t_{i}+1-z_{i}\right)\frac{e^{-\frac{\lambda }{w_{i}^{2}}}}{w_{i}^{2}\left(1-e^{-\frac{\lambda }{w_{i}^{2}}}\right)}\right]}^{2}\right.\\ \; & \left.-\sum_{i=1}^{k}\left[\left(t_{i}+1-z_{i}\right)\frac{e^{-\frac{\lambda }{w_{i}^{2}}}}{w_{i}^{4}{\left(1-e^{-\frac{\lambda }{w_{i}^{2}}}\right)}^{2}}\times \left(b_{2}-\sum_{i=1}^{k}\left(t_{i}+1-z_{i}\right)\ln\left(1-e^{-\frac{\lambda }{w_{i}^{2}}}\right)\right)\right]\right\}\\ \; & \times {\left[b_{2}-\sum_{i=1}^{k}\left(t_{i}+1-z_{i}\right)\ln\left(1-e^{-\frac{\lambda }{w_{i}^{2}}}\right)\right]}^{-2}. \end{array}$$

It cannot be determined whether (22) is negative or positive. So, log-concave method is not appropriate here. The importance sampling method [29] is adopted. (21) is rewritten as below:

(23) $$\begin{array}{cc} \pi (\lambda |data)\propto & Ga\left(c+k,d+\sum_{i=1}^{k}\frac{1}{w_{i}^{2}}\right)\times {\left[b_{1}-\sum_{i=1}^{k}\left(s_{i}+z_{i}\right)\ln\left(1-e^{-\frac{\lambda }{w_{i}^{2}}}\right)\right]}^{-(a_{1}+k_{1})}\\ \; & \times {\left[b_{2}-\sum_{i=1}^{k}\left(t_{i}+1-z_{i}\right)\ln\left(1-e^{-\frac{\lambda }{w_{i}^{2}}}\right)\right]}^{-(a_{2}+k_{2})}{\left(\prod_{i=1}^{k}\left(1-e^{-\frac{\lambda }{w_{i}^{2}}}\right)\right)}^{-1}. \end{array}$$

- **Square loss function:**

Square loss function is given below:

$$\begin{array}{cc} l_{S}\left(\hat{g}\left(\Theta \right),g\left(\Theta \right)\right) & ={\left(\hat{g}\left(\Theta \right)-g\left(\Theta \right)\right)}^{2}. \end{array}$$

where Θ means the parameter vector, g(Θ) means any function of Θ. The Bayesian estimate of any function of $\left(\theta_{1},\theta_{2},\lambda \right)$, say $g(\theta_{1},\theta_{2},\lambda )$ under square loss function can be obtained as

(24) $$\begin{array}{c} \hat{g}\left(\theta_{1},\theta_{2},\lambda \right)=\int_{0}^{\infty }\int_{0}^{\infty }\int_{0}^{\infty }g\left(\theta_{1},\theta_{2},\lambda \right)\pi \left(\theta_{1},\theta_{2},\lambda |data\right)d\theta_{1}d\theta_{2}d\lambda . \end{array}$$

The following Algorithm 3 can be used in Bayesian estimates under square loss function.
**Algorithm 3** Bayesian estimates under square loss function.Step 1: Given data (W,S,Z), generate λ from $Ga(k+c,d+\sum_{i=1}^{k}\frac{1}{w_{i}^{2}})$.Step 2: For a given λ, generate $\theta_{1}$ and $\theta_{2}$ based on (19) and (20).Step 3: Repeat Steps 1 and 2 for *M* times.Step 4: For generated ($\lambda_{\left(j\right)},\theta_{1(j)},\theta_{2(j)}$), calculate $g(\lambda_{\left(j\right)},\theta_{1(j)},\theta_{2(j)})$ and the importance weight (say $weight^{*}$),    j=1,2,⋯,M.
$$\begin{array}{c} weight_{\left(j\right)}^{*}=\frac{c_{\left(j\right)}}{\sum_{j=1}^{M}c_{\left(j\right)}}, \end{array}$$
where
$$\begin{array}{c} c_{\left(j\right)}=\frac{{\left[b_{1}-\sum_{i=1}^{k}\left(s_{i}+z_{i}\right)\ln\left(1-e^{-\frac{\lambda_{\left(j\right)}}{w_{i}^{2}}}\right)\right]}^{-(a_{1}+k_{1})}{\left[b_{2}-\sum_{i=1}^{k}\left(t_{i}+1-z_{i}\right)\ln\left(1-e^{-\frac{\lambda_{\left(j\right)}}{w_{i}^{2}}}\right)\right]}^{-(a_{2}+k_{2})}}{\prod_{i=1}^{k}\left(1-e^{-\frac{\lambda_{\left(j\right)}}{w_{i}^{2}}}\right)}. \end{array}$$Step 5: The estimate of $g(\lambda ,\theta_{1},\theta_{2})$ can be obtained by
$$\begin{array}{c} \hat{g}\left(\lambda ,\theta_{1},\theta_{2}\right)=\sum_{j=1}^{M}weight_{\left(j\right)}^{*}g\left(\lambda_{\left(j\right)},\theta_{1(j)},\theta_{2(j)}\right). \end{array}$$

- **Linex loss function:**

Linex loss function is given below:

$$\begin{array}{cc} l_{l}\left(\hat{g}\left(\Theta \right),g\left(\Theta \right)\right) & =e^{\delta \left(\hat{g}\left(\Theta \right)-g\left(\Theta \right)\right)}-\delta \left(\hat{g}\left(\Theta \right)-g\left(\Theta \right)\right)-1,\;\delta \;\mathrm{is}\;a\;\mathrm{nonzero}\;\mathrm{constant}. \end{array}$$

The Bayesian estimate of any function of $\left(\theta_{1},\theta_{2},\lambda \right)$, say $g(\theta_{1},\theta_{2},\lambda )$ under linex loss function can be obtained as

(25) $$\begin{array}{c} \hat{g}\left(\theta_{1},\theta_{2},\lambda \right)=-\frac{1}{\delta }\ln\left(\int_{0}^{\infty }\int_{0}^{\infty }\int_{0}^{\infty }\frac{\pi (\theta_{1},\theta_{2},\lambda |data)}{e^{\delta g(\theta_{1},\theta_{2},\lambda )}}d\theta_{1}d\theta_{2}d\lambda \right). \end{array}$$

The following Algorithm 4 can be used in Bayesian estimates under linex loss function.
**Algorithm 4** Bayesian estimates under linex loss function.Steps 1 to 4 are the same as those in Algorithm 3.Step 5: The estimate of $g(\lambda ,\theta_{1},\theta_{2})$ can be obtained by
$$\begin{array}{c} \hat{g}\left(\theta_{1},\theta_{2},\lambda \right)=-\frac{1}{\delta }\ln\left(\sum_{i=1}^{M}weight_{\left(j\right)}^{*}e^{-\delta g(\lambda_{\left(j\right)},\theta_{1(j)},\theta_{2(j)})}\right). \end{array}$$

To compute 100(1−α)% symmetric credible intervals of $g(\lambda ,\theta_{1},\theta_{2})$, where α means the significance level, $\left(g\left(\lambda_{\left(1\right)},\theta_{1(1)},\theta_{2(1)}\right),g\left(\lambda_{\left(2\right)},\theta_{1(2)},\theta_{2(2)}\right),\cdots ,g\left(\lambda_{\left(M\right)},\theta_{1(M)},\theta_{2(M)}\right)\right)$ are sorted in ascending order as $\left(g^{\left(1\right)},g^{\left(2\right)},\cdots ,g^{\left(M\right)}\right)$ and the corresponding weights are $\left(weight_{\left(1\right)}^{\prime },weight_{\left(2\right)}^{\prime },\cdots ,weight_{\left(M\right)}^{\prime }\right)$.

Define $sumc_{\left(\rho \right)}=\sum_{l=1}^{\rho }weight_{\left(l\right)}^{\prime }$ for l=1,2,⋯,M. When $sumc_{\left(\rho -1\right)}<\frac{\alpha }{2}$ and $sumc_{\left(\rho \right)}⩾\frac{\alpha }{2}$, record the $g^{\left(\rho \right)}$. When $sumc_{\left(\eta \right)}<1-\frac{\alpha }{2}$ and $sumc_{\left(\eta +1\right)}⩾1-\frac{\alpha }{2}$, record the $g^{\left(\eta \right)}$. Then a (1−α)% credible interval of $g(\lambda ,\theta_{1},\theta_{2})$ is

(26) $$\begin{array}{c} {}[g^{\left(\rho \right)},g^{\left(\eta \right)}]. \end{array}$$

## 6. Simulation and Data Analysis

### 6.1. Numerical Simulation

In this section, different *m*, *n*, *k* and $r_{1},r_{2},\cdots ,r_{k}$ are taken and simulation results through the methods mentioned above are displayed. We consider different censoring schemes. For the rest of the paper, the notation $\left(0_{\left(5\right)},5_{\left(5\right)},0_{\left(10\right)}\right)$ means that no unit is withdrawn for the first 5 times, 5 units are withdrawn for 5 times, and no unit is withdrawn for the last 10 times. For other notations, the meanings are similar.

Let $\theta_{1}=3,\theta_{2}=2,\lambda =2$. The average values (AVs) and mean square errors (MSEs) of MLEs are calculated by repeating the EM algorithm 1000 times. To be more practical, both the true values and $\theta_{1}^{0}=\theta_{2}^{0}=\lambda^{0}=7$ are set as the initial values in EM algorithm. The results are given in Table 1 and Table 2.

**Figure 3:**
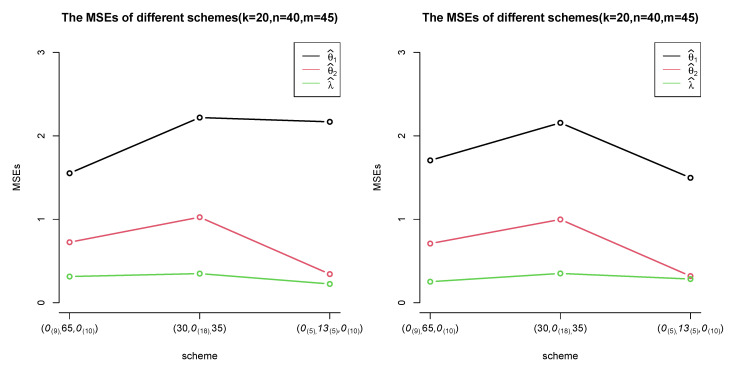
The trend of MSEs for different schemes with MLEs method (set *k* = 20, *n* = 40, *m* = 45 as an example).

In Table 1 and Table 2, the estimates of λ get closer to the true value than those of $\theta_{1}$ and $\theta_{2}$ under the same censoring scheme. The results are acceptable because λ is the same in two samples. With more information, λ gets better estimates. When considering the same sample size and failure times (*k*), there are better results if the withdrawal processes are executed in the middle rather than at the beginning and end. Besides, Figure 3 shows that a more dispersed withdrawal scheme, which means withdrawing the units for several times but not one time, yields better estimates. Comparing censoring schemes (n=20,m=25) with (n=25,m=20), the estimates of $\theta_{2}$ are better as *n* increases. The MSEs of $\hat{\theta_{2}}$ decrease.It is reasonable because if the sample size *n* increases and more information of sample B can be utilized, better estimates can be obtained. When keeping *n* and *m* fixed and increasing *k*, estimations under these schemes are better. The plots of MSEs were shown below: (take k=20,n=40,m=45 as an example).

The parameters of prior distributions are $a_{1}=2,b_{1}=1,a_{2}=1,b_{2}=2,c=3,d=2$ and linex loss constant δ=2. Then, the Bayes estimates for informative prior under square loss function (say $\hat{\theta_{1S}},\hat{\theta_{2S}},\hat{\lambda_{S}}$) and linex loss function (say $\hat{\theta_{1L}},\hat{\theta_{2L}},\hat{\lambda_{L}}$) are compared based on 1000 replications. The results are given in Table 3 and Table 4.

Table 3 and Table 4 show that MSEs are bigger and AVs are closer to the true value under linex loss function than the results under square loss function. Bayesian inference performs better than MLEs in terms of MSEs. However, Bayesian estimators mostly underestimate the true parameter values and MLEs do not show this pattern. In the schemes with larger sample sizes, MLEs get better AVs but bigger MSEs than Bayesian estimators. The tables also reveal that MSEs become small with *k* increases. As true value increases, the methods mentioned above all show bigger MSEs, which means that the results are more dispersed.

Next, we compare Bayes estimates for non-informative prior and informative prior. According to [30], let hyperparameters $a_{1}=0.0001,b_{1}=0.0001,a_{2}=0.0001,b_{2}=0.0001,$c=0.0001,d=0.0001. The patterns of different schemes are similar to those mentioned above. Observing Table 5 and Table 6, it is found that if the samples have more units but the failure times are relatively less, Bayesian estimators with informative priors perform better than those with non-informative priors. When the failure times are relatively more, in another word, there are more observed data even the sample sizes are small, and the results with these two methods have little difference. In addition, the results are closer to the true value under square loss function. In a word, Bayesian estimation with informative prior under square loss function performs best among the methods discussed.

Besides the point estimates, Bootstrap-p, Bootstrap-t, and Bayesian methods are used to obtain the 90% confidence/credible intervals. In Table 7 and Table 8, the average lengths (ALs) and coverage percentages (CPs) are calculated based on 1000 replications. In Bootstrap methods, boot-time is set as 1000 (N=1000). Here, IP means under informative prior density and NIP means under non-informative prior density.

Table 7 displays the ALs and CPs of confidence intervals with Bootstrap-p and Bootstrap-t methods. The contrast between Bootstrap-p and Bootstrap-t indicates that CPs are similar but ALs of Bootstrap-t are wider than those of Bootstrap-p. Therefore, the Bootstrap-p method is more appropriate to get the confidence intervals.

**Figure 4:**
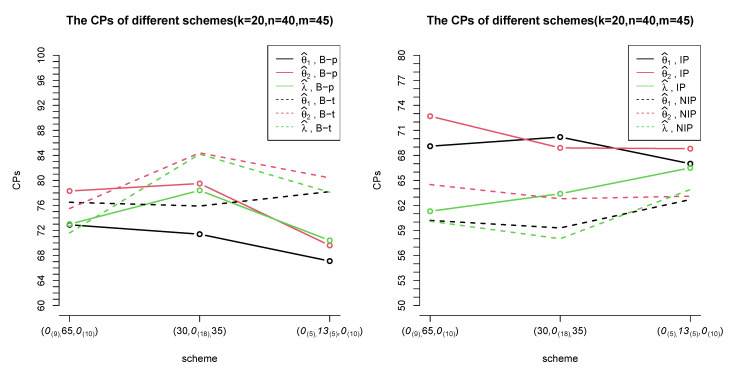
The trend of CPs for different schemes with four methods (set *k* = 20, *n* = 40, *m* = 45 as an example).

Table 8 shows the ALs and CPs of credible intervals under informative prior density and non-informative prior density. The contrast indicates that ALs of NIP tend to be longer than those of IP but CPs are less than those of IP. Obviously, IP performs better than NIP. Besides, for a fixed scheme, $\theta_{2}$ has the best estimates of credible intervals. Figure 4 displays the contrast of CPs among different methods which also indicates that Bootstrap-p and Bayes method with informative prior are more suitable for interval estimates. Compared to Bootstrap methods, Bayesian method yields better results of credible intervals. In the condition of large *k*, CPs increase a lot with both the Bayesian method and Bootstrap methods. When there are sufficient units, choosing the Bayesian method with informative priors is better.

The plots of CPs were shown below: (take k=20,n=40,m=45 as an example).

### 6.2. Real Data Analysis

In this part, we analyze one application in the coating weights with two real data sets and apply the approaches put forward in the sections above. The data sets are from ALAF (formerly called Aluminium Africa Limited) industry, Tanzania, which contain the coating weights (mg/m${}^{2}$) by chemical procedure on the top center side (TCS) and by chemical procedure on the bottom center side (BCS). For each data set, there are 72 observations. The data were also analyzed by [11]. The data are shown below: 

**Data set 1: (TCS)**

36.8, 47.2, 35.6, 36.7, 55.8, 58.7, 42.3, 37.8, 55.4, 45.2, 31.8, 48.3, 45.3, 48.5, 52.8, 45.4, 49.8,

48.2, 54.5, 50.1, 48.4, 44.2, 41.2, 47.2, 39.1, 40.7, 40.3, 41.2, 30.4, 42.8, 38.9, 34.0, 33.2, 56.8,

52.6, 40.5, 40.6, 45.8, 58.9, 28.7, 37.3, 36.8, 40.2, 58.2, 59.2, 42.8, 46.3, 61.2, 58.4, 38.5, 34.2,

41.3, 42.6, 43.1, 42.3, 54.2, 44.9, 42.8, 47.1, 38.9, 42.8, 29.4, 32.7, 40.1, 33.2, 31.6, 36.2, 33.6,

32.9, 34.5, 33.7, 39.9

**Data set 2: (BCS)**

45.5, 37.5, 44.3, 43.6, 47.1, 52.9, 53.6, 42.9, 40.6, 34.1, 42.6, 38.9, 35.2, 40.8, 41.8, 49.3, 38.2,

48.2, 44.0, 30.4, 62.3, 39.5, 39.6, 32.8, 48.1, 56.0, 47.9, 39.6, 44.0, 30.9, 36.6, 40.2, 50.3, 34.3,

54.6, 52.7, 44.2, 38.9, 31.5, 39.6, 43.9, 41.8, 42.8, 33.8, 40.2, 41.8, 39.6, 24.8, 28.9, 54.1, 44.1,

52.7, 51.5, 54.2, 53.1, 43.9, 40.8, 55.9, 57.2, 58.9, 40.8, 44.7, 52.4, 43.8, 44.2, 40.7, 44.0, 46.3,

41.9, 43.6, 44.9, 53.6

**Figure 5:**
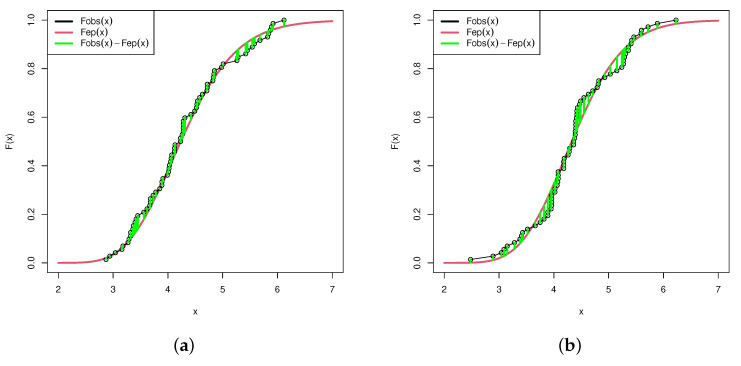
The IERD fitness of data sets. Fobs(x) means the empirical cumulative distribution function of data set. Fep(x) means the fitted distribution function of data set. (**a**) The fitness of data set 1. (**b**) The fitness of data set 2.

For convenience, the data sets are divided by 10. First, to verify that IERD is suitable for the data sets, we fit it for each data set and have Kolmogorov-Smirnov(K-S) distance test. By calculating the largest difference value of empirical cumulative distribtuion functions and the fitted distribution functions and comparing that value with the 95% critical value, we find the data sets can be fitted well. The results are shown in Table 9:

K-S distances are less than 95% critical value, so the IERD fits well for both data sets. Figure 5 shows the fitness of the data sets separately. Then, the likelihood ratio test is used to test if the scale parameters can be considered as the same value. $H_{0}:\lambda_{1}=\lambda_{2}$. The *p*-value is calculated to be 94.3%. Obviously, the null hypothesis cannot be rejected. The two scale parameters can be considered as the same. Based on the null hypothesis, the MLEs are obtained as $\hat{\theta_{1}}=15.55,\hat{\theta_{2}}=14.87,\hat{\lambda }=57.08$.

Use the complete data above and generate observed data for the following three censoring schemes, $\left(0_{\left(18\right)},2_{\left(36\right)},0_{\left(18\right)}\right)$, $\left(0_{\left(35\right)},36_{\left(2\right)},0_{\left(35\right)}\right)$, and $\left(36,0_{\left(70\right)},36\right)$. Take MLEs for complete data as the initial values of EM algorithm. Then, the AVs and MSEs of MLEs can be obtained in Table 10.

**Figure 6:**
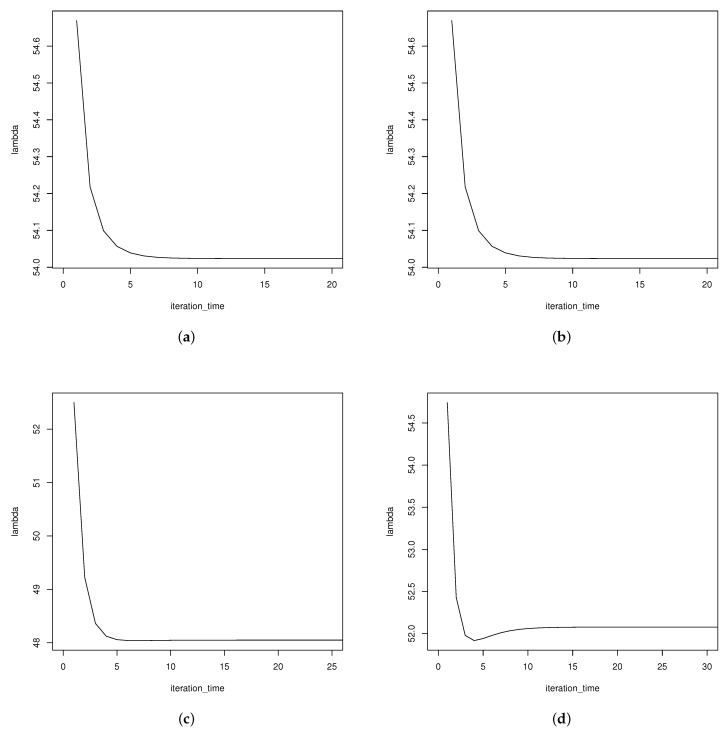
(**a**,**b**) The iterations for two different initial guesses of lambda, 57.07 and 70, under $\left(0_{\left(18\right)},2_{\left(36\right)},0_{\left(18\right)}\right)$. (**c**,**d**) The iterations for two different initial guesses of lambda, 57.07 and 70, under $\left(36,0_{\left(70\right)},36\right)$.

To verify the stablitity of iteration, we change the initial guesses and plot the trend of the estimates. The results are shown in Figure 6. The iteration times are 15 times in (a), 23 times in (b) and (c), and 26 times in (d). It is observed that with the same initial guess of λ, the more dispersed scheme need less iteration times. When the initial guesses are not close to the true value, the iteration times will increase but the processes are still stable.

In this case, we cannot get the informative priors, so all Bayesian estimates are based on non-informative priors. Table 11 and Table 12 record the results of Bayesian method with non-informative priors. The 90% confidence/credible intervals with Bootstrap methods and Bayes estimates for non-informative prior are displayed in Table 13.

From the real data, some facts are displayed. Bayesian point estimates for non-informative prior under square loss function are higher than those under linex function. Besides, the first scheme $\left(0_{\left(18\right)},2_{\left(36\right)},0_{\left(18\right)}\right)$ corresponds to higher estimates in point estimations and shorter interval lengths in interval estimations. Table 13 reveals that Bayesian inference under non-informative priors yields shorter interval lengths than Bootstrap-p and Bootstrap-t methods. More dispersed schemes and Bayesian inference are preferred in real data analysis.

## 7. Conclusions

In this article, we studied two samples following inverted exponentiated Rayleigh distribution under joint progressively type-II censoring scheme. It was supposed that the shape parameters were different but that the scale parameters were the same. The expectation-maximization algorithm was applied to obtain the estimates of MLEs. The performance of MLEs and Bayesian estimators for non-informative prior and informative prior was compared. Bootstrap-p, Bootstrap-t, and Bayesian methods were used in intervals estimations. Importance sampling technique was introduced when calculating Bayesian estimates. The contrast between estimates under square loss function and linex loss function was also studied.

During the point estimation process, Bayesian inference under informative priors turned out to be the best method and many patterns of censoring scheme were concluded. To study the confidence intervals, Bootstrap methods were applied and evaluated. Besides, observed Fisher information matrix played a key role in getting Bootstrap-t intervals based on the missing value principle.

In the future, the methods can be extended to many other distributions such as multivariate Gaussian distribution with zero mean. Ref. [31] proposed a total Bregman divergence-based matrix information geometry (TBD-MIG) detector and applied it to detect targets emerged into nonhomogeneous clutter. We are still doing more work the situations where the scale parameters are different and the samples are not independent.

## Figures and Tables

**Table 1:** AVs and MSEs of MLEs when the true values is set as the initial values.

*k*	*n*	*m*	Scheme	$\hat{\theta_{1}}$			$\hat{\theta_{2}}$			$\hat{\lambda }$	
				**AV**	**MSE**		**AV**	**MSE**		**AV**	**MSE**
20	20	25	($0_{\left(9\right)},25,0_{\left(10\right)}$)	2.778	0.960		1.951	0.511		1.866	0.167
			($10,0_{\left(18\right)},15$)	2.330	1.168		1.525	0.574		1.606	0.269
			($0_{\left(5\right)},5_{\left(5\right)},0_{\left(10\right)}$)	3.026	1.277		1.835	0.482		1.878	0.161
	25	20	($0_{\left(9\right)},25,0_{\left(10\right)}$)	2.959	0.778		1.878	0.275		1.896	0.127
			($10,0_{\left(18\right)},15$)	2.376	0.991		1.538	0.351		1.602	0.196
			($0_{\left(5\right)},5_{\left(5\right)},0_{\left(10\right)}$)	2.303	0.949		1.470	0.402		1.632	0.177
	40	45	($0_{\left(9\right)},65,0_{\left(10\right)}$)	2.181	1.552		1.598	0.725		1.561	0.314
			($30,0_{\left(18\right)},35$)	2.160	2.219		1.624	1.025		1.435	0.349
			($0_{\left(5\right)},13_{\left(5\right)},0_{\left(10\right)}$)	2.168	1.276		1.684	0.343		1.887	0.225
30	20	25	($0_{\left(14\right)},15,0_{\left(15\right)}$)	3.246	1.021		2.538	0.516		2.128	0.116
			($5,0_{\left(28\right)},10$)	3.210	0.801		2.057	0.213		2.030	0.075
			($0_{\left(12\right)},3_{\left(5\right)},0_{\left(13\right)}$)	3.117	1.119		2.148	0.290		2.196	0.115
	40	45	($0_{\left(14\right)},55,0_{\left(15\right)}$)	2.561	0.871		1.553	0.322		1.684	0.129
			($27,0_{\left(28\right)},28$)	2.216	1.350		1.512	0.618		1.600	0.277
			($0_{\left(9\right)},5_{\left(11\right)},0_{\left(10\right)}$)	2.679	1.132		1.590	0.402		1.728	0.117
40	40	45	($0_{\left(19\right)},45,0_{\left(20\right)}$)	3.076	0.799		1.870	0.252		1.927	0.111
			($25,0_{\left(38\right)},20$)	2.769	0.625		1.526	0.307		1.659	0.149
			($0_{\left(17\right)},9_{\left(5\right)},0_{\left(18\right)}$)	2.859	0.672		1.823	0.193		1.871	0.075

**Table 2:** AVs and MSEs of MLEs when 7 is set as the initial values.

*k*	*n*	*m*	Scheme	$\hat{\theta_{1}}$			$\hat{\theta_{2}}$			$\hat{\lambda }$	
				**AV**	**MSE**		**AV**	**MSE**		**AV**	**MSE**
20	20	25	($0_{\left(9\right)},25,0_{\left(10\right)}$)	2.931	1.261		1.813	0.264		1.844	0.103
			($10,0_{\left(18\right)},15$)	2.168	0.967		1.395	0.472		1.588	0.227
			($0_{\left(5\right)},5_{\left(5\right)},0_{\left(10\right)}$)	2.977	1.078		1.821	0.502		1.883	0.146
	25	20	($0_{\left(9\right)},25,0_{\left(10\right)}$)	2.846	1.098		1.903	0.394		1.868	0.139
			($10,0_{\left(18\right)},15$)	2.232	0.938		1.503	0.348		1.637	0.177
			($0_{\left(5\right)},5_{\left(5\right)},0_{\left(10\right)}$)	2.901	1.005		1.919	0.356		1.881	0.120
	40	45	($0_{\left(9\right)},65,0_{\left(10\right)}$)	1.760	1.706		1.221	0.709		1.526	0.252
			($30,0_{\left(18\right)},35$)	1.563	2.156		1.027	0.998		1.427	0.351
			($0_{\left(5\right)},13_{\left(5\right)},0_{\left(10\right)}$)	2.141	1.497		1.467	0.319		1.536	0.284
30	20	25	($0_{\left(14\right)},15,0_{\left(15\right)}$)	3.102	0.751		2.234	0.220		1.963	0.066
			($5,0_{\left(28\right)},10$)	3.111	0.514		1.999	0.193		1.989	0.069
			($0_{\left(12\right)},3_{\left(5\right)},0_{\left(13\right)}$)	2.902	0.618		2.056	0.147		2.008	0.061
	40	45	($0_{\left(14\right)},55,0_{\left(15\right)}$)	2.077	0.959		1.458	0.455		1.594	0.194
			($27,0_{\left(28\right)},28$)	1.824	1.520		1.243	0.640		1.490	0.286
			($0_{\left(9\right)},5_{\left(11\right)},0_{\left(10\right)}$)	2.279	1.135		1.549	0.303		1.647	0.155
40	40	45	($0_{\left(19\right)},45,0_{\left(20\right)}$)	2.03	0.824		1.860	0.237		1.863	0.077
			($25,0_{\left(38\right)},20$)	2.335	0.597		1.522	0.301		1.634	0.161
			($0_{\left(17\right)},9_{\left(5\right)},0_{\left(18\right)}$)	2.952	0.536		1.917	0.205		1.933	0.094

**Table 3:** AVs and MSEs of Bayesian estimates for informative prior under square loss function based on 1000 replications.

*k*	*n*	*m*	Scheme	${\hat{\theta_{1}}}_{S}$			${\hat{\theta_{2}}}_{S}$			${\hat{\lambda }}_{S}$	
				**AV**	**MSE**		**AV**	**MSE**		**AV**	**MSE**
20	20	25	($0_{\left(9\right)},25,0_{\left(10\right)}$)	2.779	0.519		1.712	0.233		1.751	0.250
			($10,0_{\left(18\right)},15$)	2.748	0.523		1.643	0.270		1.679	0.314
			($0_{\left(5\right)},5_{\left(5\right)},0_{\left(10\right)}$)	2.712	0.394		1.781	0.207		1.768	0.230
	25	20	($0_{\left(9\right)},25,0_{\left(10\right)}$)	2.748	0.437		1.671	0.243		1.749	0.251
			($10,0_{\left(18\right)},15$)	2.729	0.524		1.696	0.290		1.783	0.311
			($0_{\left(5\right)},5_{\left(5\right)},0_{\left(10\right)}$)	2.817	0.486		1.826	0.172		1.794	0.211
	40	45	($0_{\left(9\right)},65,0_{\left(10\right)}$)	2.480	1.220		1.566	0.499		1.591	0.399
			($30,0_{\left(18\right)},35$)	2.381	1.420		1.518	0.697		1.577	0.546
			($0_{\left(5\right)},13_{\left(5\right)},0_{\left(10\right)}$)	2.614	1.034		1.618	0.463		1.612	0.374
30	20	25	($0_{\left(14\right)},15,0_{\left(15\right)}$)	2.831	0.355		1.867	0.168		1.878	0.218
			($5,0_{\left(28\right)},10$)	2.769	0.345		1.803	0.171		1.835	0.253
			($0_{\left(12\right)},3_{\left(5\right)},0_{\left(13\right)}$)	2.855	0.334		1.894	0.143		1.873	0.219
	40	45	($0_{\left(14\right)},55,0_{\left(15\right)}$)	2.421	1.083		1.634	0.406		1.680	0.406
			($27,0_{\left(28\right)},28$)	2.334	1.064		1.608	0.435		1.625	0.479
			($0_{\left(9\right)},5_{\left(11\right)},0_{\left(10\right)}$)	2.679	1.070		1.717	0.423		1.741	0.456
40	40	45	($0_{\left(19\right)},45,0_{\left(20\right)}$)	2.651	0.830		1.837	0.289		1.705	0.374
			($25,0_{\left(38\right)},20$)	2.684	0.654		1.674	0.259		1.693	0.387
			($0_{\left(17\right)},9_{\left(5\right)},0_{\left(18\right)}$)	2.641	0.836		1.830	0.286		1.791	0.390

**Table 4:** AVs and MSEs of Bayesian estimates for informative prior under linex loss function based on 1000 replications.

*k*	*n*	*m*	Scheme	${\hat{\theta_{1}}}_{L}$			${\hat{\theta_{2}}}_{L}$			${\hat{\lambda }}_{L}$	
				**AV**	**MSE**		**AV**	**MSE**		**AV**	**MSE**
20	20	25	($0_{\left(9\right)},25,0_{\left(10\right)}$)	2.568	0.754		1.678	0.297		1.616	0.279
			($10,0_{\left(18\right)},15$)	2.695	0.842		1.712	0.349		1.647	0.346
			($0_{\left(5\right)},5_{\left(5\right)},0_{\left(10\right)}$)	2.583	0.595		1.689	0.265		1.533	0.259
	25	20	($0_{\left(9\right)},25,0_{\left(10\right)}$)	2.673	0.636		1.740	0.320		1.612	0.281
			($10,0_{\left(18\right)},15$)	2.683	0.762		1.782	0.372		1.647	0.347
			($0_{\left(5\right)},5_{\left(5\right)},0_{\left(10\right)}$)	2.659	0.673		1.685	0.212		1.656	0.243
	40	45	($0_{\left(9\right)},65,0_{\left(10\right)}$)	2.371	1.425		1.516	0.554		1.580	0.412
			($30,0_{\left(18\right)},35$)	2.291	1.825		1.472	0.761		1.566	0.562
			($0_{\left(5\right)},13_{\left(5\right)},0_{\left(10\right)}$)	2.582	1.241		1.662	0.517		1.600	0.388
30	20	25	($0_{\left(14\right)},15,0_{\left(15\right)}$)	2.762	0.510		1.843	0.194		1.849	0.242
			($5,0_{\left(28\right)},10$)	2.738	0.505		1.889	0.208		1.808	0.278
			($0_{\left(12\right)},3_{\left(5\right)},0_{\left(13\right)}$)	2.797	0.458		1.871	0.163		1.845	0.242
	40	45	($0_{\left(14\right)},55,0_{\left(15\right)}$)	2.345	1.232		1.598	0.443		1.672	0.415
			($27,0_{\left(28\right)},28$)	2.256	1.205		1.568	0.477		1.617	0.490
			($0_{\left(9\right)},5_{\left(11\right)},0_{\left(10\right)}$)	2.412	1.298		1.682	0.459		1.734	0.464
40	40	45	($0_{\left(19\right)},45,0_{\left(20\right)}$)	2.582	0.945		1.605	0.317		1.698	0.382
			($25,0_{\left(38\right)},20$)	2.504	0.759		1.531	0.290		1.685	0.397
			($0_{\left(17\right)},9_{\left(5\right)},0_{\left(18\right)}$)	2.579	0.941		1.698	0.313		1.685	0.397

**Table 5:** AVs and MSEs of Bayesian estimates for non-informative prior under square loss function based on 1000 replications.

*k*	*n*	*m*	Scheme	${\hat{\theta_{1}}}_{\mathrm{NS}}$			${\hat{\theta_{2}}}_{\mathrm{NS}}$			${\hat{\lambda }}_{\mathrm{NS}}$	
				**AV**	**MSE**		**AV**	**MSE**		**AV**	**MSE**
20	20	25	($0_{\left(9\right)},25,0_{\left(10\right)}$)	2.617	0.677		2.060	0.352		1.556	0.252
			($10,0_{\left(18\right)},15$)	2.437	0.710		1.908	0.365		1.462	0.340
			($0_{\left(5\right)},5_{\left(5\right)},0_{\left(10\right)}$)	2.756	0.564		2.162	0.458		1.562	0.247
	25	20	($0_{\left(9\right)},25,0_{\left(10\right)}$)	2.809	0.672		1.992	0.360		1.559	0.256
			($10,0_{\left(18\right)},15$)	2.655	0.724		1.833	0.287		1.479	0.325
			($0_{\left(5\right)},5_{\left(5\right)},0_{\left(10\right)}$)	2.986	0.966		2.154	0.343		1.618	0.214
	40	45	($0_{\left(9\right)},65,0_{\left(10\right)}$)	2.195	1.688		1.421	0.487		1.345	0.461
			($30,0_{\left(18\right)},35$)	2.094	1.717		1.337	0.716		1.329	0.621
			($0_{\left(5\right)},13_{\left(5\right)},0_{\left(10\right)}$)	2.265	1.359		1.524	0.492		1.379	0.420
30	20	25	($0_{\left(14\right)},15,0_{\left(15\right)}$)	2.792	0.372		2.160	0.283		1.688	0.216
			($5,0_{\left(28\right)},10$)	2.716	0.389		2.084	0.270		1.646	0.254
			($0_{\left(12\right)},3_{\left(5\right)},0_{\left(13\right)}$)	2.832	0.386		2.199	0.313		1.789	0.214
	40	45	($0_{\left(14\right)},55,0_{\left(15\right)}$)	2.333	1.310		1.517	0.355		1.551	0.445
			($27,0_{\left(28\right)},28$)	2.326	1.311		1.456	0.419		1.493	0.524
			($0_{\left(9\right)},5_{\left(11\right)},0_{\left(10\right)}$)	2.483	1.374		1.666	0.393		1.611	0.499
40	40	45	($0_{\left(19\right)},45,0_{\left(20\right)}$)	2.485	0.972		1.704	0.257		1.683	0.400
			($25,0_{\left(38\right)},20$)	2.452	0.713		1.671	0.217		1.680	0.408
			($0_{\left(17\right)},9_{\left(5\right)},0_{\left(18\right)}$)	2.681	0.960		1.806	0.242		1.777	0.409

**Table 6:** AVs and MSEs of Bayesian estimates for non-informative prior under linex loss function based on 1000 replications.

*k*	*n*	*m*	Scheme	${\hat{\theta_{1}}}_{\mathrm{NL}}$			${\hat{\theta_{2}}}_{\mathrm{NL}}$			${\hat{\lambda }}_{\mathrm{NL}}$	
				**AV**	**MSE**		**AV**	**MSE**		**AV**	**MSE**
20	20	25	($0_{\left(9\right)},25,0_{\left(10\right)}$)	2.550	0.902		1.835	0.272		1.523	0.281
			($10,0_{\left(18\right)},15$)	2.456	0.998		1.704	0.339		1.432	0.373
			($0_{\left(5\right)},5_{\left(5\right)},0_{\left(10\right)}$)	2.577	0.686		1.903	0.289		1.532	0.273
	25	20	($0_{\left(9\right)},25,0_{\left(10\right)}$)	2.509	0.776		1.786	0.300		1.527	0.285
			($10,0_{\left(18\right)},15$)	2.459	0.932		1.665	0.314		1.547	0.359
			($0_{\left(5\right)},5_{\left(5\right)},0_{\left(10\right)}$)	2.510	0.801		1.950	0.236		1.583	0.239
	40	45	($0_{\left(9\right)},65,0_{\left(10\right)}$)	2.179	1.925		1.353	0.552		1.334	0.476
			($30,0_{\left(18\right)},35$)	2.150	1.962		1.269	0.804		1.316	0.641
			($0_{\left(5\right)},13_{\left(5\right)},0_{\left(10\right)}$)	2.283	1.582		1.442	0.532		1.467	0.435
30	20	25	($0_{\left(14\right)},15,0_{\left(15\right)}$)	2.606	0.507		1.990	0.211		1.662	0.236
			($5,0_{\left(28\right)},10$)	2.582	0.522		1.930	0.221		1.623	0.275
			($0_{\left(12\right)},3_{\left(5\right)},0_{\left(13\right)}$)	2.764	0.465		2.033	0.215		1.765	0.233
	40	45	($0_{\left(14\right)},55,0_{\left(15\right)}$)	2.354	1.456		1.570	0.393		1.543	0.455
			($27,0_{\left(28\right)},28$)	2.284	1.472		1.502	0.466		1.484	0.536
			($0_{\left(9\right)},5_{\left(11\right)},0_{\left(10\right)}$)	2.416	1.522		1.624	0.434		1.504	0.508
40	40	45	($0_{\left(19\right)},45,0_{\left(20\right)}$)	2.418	1.093		1.666	0.283		1.677	0.408
			($25,0_{\left(38\right)},20$)	2.371	0.827		1.623	0.243		1.673	0.417
			($0_{\left(17\right)},9_{\left(5\right)},0_{\left(18\right)}$)	2.717	1.074		1.770	0.269		1.771	0.416

**Table 7:** ALs and CPs of 90% confidence intervals with Boostrap-p and Bootstrap-t methods based on 1000 replications.

Scheme	*Parameter*	Bootstrap-p			Bootstrap-t	
		**AL**	**CP(%)**		**AL**	**CP(%)**
k=20,n=20,m=25	$\theta_{1}$	2.721	87.8		3.353	91.8
scheme = $\left(0_{\left(9\right)},25,0_{\left(10\right)}\right)$	$\theta_{2}$	1.489	91.9		1.104	88.3
	λ	1.313	87.6		1.291	87.5
k=20,n=20,m=25	$\theta_{1}$	1.642	72.5		1.472	73.5
scheme = $\left(10,0_{\left(18\right)},15\right)$	$\theta_{2}$	1.221	81.2		1.741	81.7
	λ	0.706	73.2		0.867	72.7
k=20,n=20,m=25	$\theta_{1}$	2.823	75.5		3.407	77.8
scheme = $\left(0_{\left(5\right)},5_{\left(5\right)},0_{\left(10\right)}\right)$	$\theta_{2}$	1.489	77.1		2.884	78.3
	λ	1.313	77.6		0.291	78.1
k=20,n=25,m=20	$\theta_{1}$	2.900	98.8		3.339	93.9
scheme = $\left(0_{\left(9\right)},25,0_{\left(10\right)}\right)$	$\theta_{2}$	1.505	93.4		1.199	90.1
	λ	1.004	93.2		1.051	95.6
k=20,n=25,m=20	$\theta_{1}$	1.667	74.3		4.027	74.3
scheme = $\left(10,0_{\left(18\right)},15\right)$	$\theta_{2}$	1.221	87.2		2.138	88.9
	λ	0.707	75.6		0.685	77.5
k=20,n=25,m=20	$\theta_{1}$	2.629	83.8		2.282	87.3
scheme = $\left(0_{\left(5\right)},5_{\left(5\right)},0_{\left(10\right)}\right)$	$\theta_{2}$	2.180	74.3		2.627	70.2
	λ	1.871	78.9		2.197	84.3
k=20,n=40,m=45	$\theta_{1}$	1.177	72.9		3.268	76.5
scheme = $\left(0_{\left(9\right)},65,0_{\left(10\right)}\right)$	$\theta_{2}$	1.016	78.3		0.919	75.5
	λ	0.572	73.0		0.888	71.6
k=20,n=40,m=45	$\theta_{1}$	1.003	71.4		1.163	75.9
scheme = $\left(30,0_{\left(18\right)},35\right)$	$\theta_{2}$	0.887	79.5		1.245	84.4
	λ	0.493	78.4		2.705	84.2
k=20,n=40,m=45	$\theta_{1}$	1.281	67.1		2.255	78.2
scheme = $\left(0_{\left(5\right)},13_{\left(5\right)},0_{\left(10\right)}\right)$	$\theta_{2}$	1.132	69.6		2.475	80.4
	λ	0.695	70.4		0.670	78.1
k=30,n=20,m=25	$\theta_{1}$	1.500	71.6		1.566	82.9
scheme = $\left(0_{\left(14\right)},15,0_{\left(15\right)}\right)$	$\theta_{2}$	1.009	76.2		2.370	86.2
	λ	0.621	79.3		0.867	75.5
k=30,n=20,m=25	$\theta_{1}$	0.802	77.1		2.343	80.8
scheme = $\left(5,0_{\left(28\right)},10\right)$	$\theta_{2}$	1.860	78.5		2.328	87.3
	λ	1.019	80.5		1.538	89.2
k=30,n=20,m=25	$\theta_{1}$	1.060	84.1		1.813	85.4
scheme = $\left(0_{\left(12\right)},3_{\left(5\right)},0_{\left(13\right)}\right)$	$\theta_{2}$	1.946	87.2		0.917	80.5
	λ	2.237	80.3		0.890	74.2

**Table 8:** ALs and CPs of 90% symmetric credible intervals based on 1000 replications.

Scheme	*Parameter*	IP			NIP	
		**AL**	**CP(%)**		**AL**	**CP(%)**
k=20,n=20,m=25	$\theta_{1}$	1.111	78.7		1.117	77.5
scheme = $\left(0_{\left(9\right)},25,0_{\left(10\right)}\right)$	$\theta_{2}$	0.797	81.8		0.897	79.5
	λ	0.370	74.3		0.416	72.7
k=20,n=20,m=25	$\theta_{1}$	1.012	69.2		1.055	63.7
scheme = $\left(10,0_{\left(18\right)},15\right)$	$\theta_{2}$	0.773	79.8		0.864	72.4
	λ	0.358	65.0		0.393	60.6
k=20,n=20,m=25	$\theta_{1}$	1.127	87.0		1.211	84.3
scheme = $\left(0_{\left(5\right)},5_{\left(5\right)},0_{\left(10\right)}\right)$	$\theta_{2}$	0.843	89.2		0.964	87.5
	λ	0.359	81.3		0.417	78.1
k=20,n=25,m=20	$\theta_{1}$	1.239	87.7		1.301	87.7
scheme = $\left(0_{\left(9\right)},25,0_{\left(10\right)}\right)$	$\theta_{2}$	0.794	88.8		0.850	84.3
	λ	0.374	89.3		0.428	82.8
k=20,n=25,m=20	$\theta_{1}$	1.213	84.5		1.271	78.4
scheme = $\left(10,0_{\left(18\right)},15\right)$	$\theta_{2}$	0.751	87.3		0.806	81.7
	λ	0.381	79.7		0.423	71.1
k=20,n=25,m=20	$\theta_{1}$	1.385	87.2		1.472	87.0
scheme = $\left(0_{\left(5\right)},5_{\left(5\right)},0_{\left(10\right)}\right)$	$\theta_{2}$	0.840	89.2		0.872	83.4
	λ	0.388	79.3		0.461	76.8
k=20,n=40,m=45	$\theta_{1}$	0.530	69.1		0.538	60.2
scheme = $\left(0_{\left(9\right)},65,0_{\left(10\right)}\right)$	$\theta_{2}$	0.376	72.7		0.427	64.5
	λ	0.190	61.3		0.195	60.1
k=20,n=40,m=45	$\theta_{1}$	0.525	70.2		0.544	59.3
scheme = $\left(30,0_{\left(18\right)},35\right)$	$\theta_{2}$	0.393	68.9		0.462	62.8
	λ	0.207	63.4		0.227	58.0
k=20,n=40,m=45	$\theta_{1}$	0.556	67.0		0.596	62.7
scheme = $\left(0_{\left(5\right)},13_{\left(5\right)},0_{\left(10\right)}\right)$	$\theta_{2}$	0.412	68.8		0.439	63.1
	λ	0.194	66.5		0.202	63.9
k=30,n=20,m=25	$\theta_{1}$	1.051	91.8		1.115	88.9
scheme = $\left(0_{\left(14\right)},15,0_{\left(15\right)}\right)$	$\theta_{2}$	0.781	92.4		0.825	91.2
	λ	0.344	84.4		0.389	83.2
k=30,n=20,m=25	$\theta_{1}$	0.943	83.8		1.019	82.5
scheme = $\left(5,0_{\left(28\right)},10\right)$	$\theta_{2}$	0.736	86.0		0.776	81.6
	λ	0.320	81.6		0.373	80.9
k=30,n=20,m=25	$\theta_{1}$	0.999	93.2		1.081	90.4
scheme = $\left(0_{\left(12\right)},3_{\left(5\right)},0_{\left(13\right)}\right)$	$\theta_{2}$	0.766	94.3		0.795	92.0
	λ	0.323	85.6		0.377	82.3

**Table 9:** The fitting results of the two data sets.

Data Set	$\hat{\theta }$	$\hat{\lambda }$	K-S Distance	95% Critical Value
data set 1	13.18	53.30	0.0612	0.1603
data set 2	18.22	61.56	0.0871	0.1603

**Table 10:** Maximum likelihood estimates under three schemes.

Scheme	$\hat{\theta_{1}}$		$\hat{\theta_{2}}$		$\hat{\lambda }$
$\left(0_{\left(18\right)},2_{\left(36\right)},0_{\left(18\right)}\right)$	14.73		13.97		54.15
$\left(0_{\left(35\right)},36_{\left(2\right)},0_{\left(35\right)}\right)$	16.78		12.91		57.23
$\left(36,0_{\left(70\right)},36\right)$	13.50		12.76		55.51

**Table 11:** Bayes estimates for non-informative prior under square loss function.

Scheme	$\hat{\theta_{1}}$		$\hat{\theta_{2}}$		$\hat{\lambda }$
$\left(0_{\left(18\right)},2_{\left(36\right)},0_{\left(18\right)}\right)$	14.40		13.47		54.73
$\left(0_{\left(35\right)},36_{\left(2\right)},0_{\left(35\right)}\right)$	13.55		11.97		54.72
$\left(36,0_{\left(70\right)},36\right)$	13.29		10.99		54.68

**Table 12:** Bayes estimates for non-informative prior under linex loss function.

Scheme	$\hat{\theta_{1}}$		$\hat{\theta_{2}}$		$\hat{\lambda }$
$\left(0_{\left(18\right)},2_{\left(36\right)},0_{\left(18\right)}\right)$	14.23		13.38		53.56
$\left(0_{\left(35\right)},36_{\left(2\right)},0_{\left(35\right)}\right)$	13.41		11.86		53.50
$\left(36,0_{\left(70\right)},36\right)$	13.16		10.89		53.34

**Table 13:** The interval estimates with three methods. LB means lower bound and UB means upper bound.

Scheme	Parameter	Bootstrap-p		Bootstrap-t		NIP	
		LB	UB	LB	UB	LB	UB
	$\theta_{1}$	12.26	17.57	11.97	18.02	12.45	18.51
$\left(0_{\left(18\right)},2_{\left(36\right)},0_{\left(18\right)}\right)$	$\theta_{2}$	10.23	17.05	9.91	18.20	11.26	16.78
	λ	53.33	61.59	53.06	62.30	53.51	60.13
	$\theta_{1}$	11.37	18.41	11.88	19.76	12.43	19.17
$\left(0_{\left(35\right)},36_{\left(2\right)},0_{\left(35\right)}\right)$	$\theta_{2}$	9.36	17.29	9.40	18.55	10.23	17.96
	λ	51.60	60.64	50.05	61.23	53.30	61.18
	$\theta_{1}$	13.27	20.74	12.81	20.07	12.25	19.46
$\left(36,0_{\left(70\right)},36\right)$	$\theta_{2}$	11.69	19.80	11.09	19.08	12.72	20.79
	λ	53.53	65.16	52.33	64.16	51.41	62.11

## Data Availability

The data presented in this study are openly available in [11].

## Appendix A

The elements of the Fisher information matrix are expressed as follows:

$-E\left(\frac{\partial^{2}\ln f_{IERD}\left(x;\theta_{1},\lambda \right)}{\partial \theta_{1}^{2}}\right)=\frac{1}{\theta_{1}^{2}}$

$-E\left(\frac{\partial^{2}\ln f_{IERD}\left(x;\theta_{1},\lambda \right)}{\partial \theta_{1}\partial \lambda }\right)=-\int_{0}^{\infty }2\theta_{1}\lambda x^{-5}e^{-\frac{2\lambda }{x^{2}}}{\left(1-e^{-\frac{\lambda }{x^{2}}}\right)}^{\theta_{1}-2}dx$

$-E\left(\frac{\partial^{2}\ln f_{IERD}\left(x;\theta_{1},\lambda \right)}{\partial \lambda^{2}}\right)=\frac{1}{\lambda^{2}}-\left(\theta_{1}-1\right)\int_{0}^{\infty }2\theta_{1}\lambda x^{-7}e^{-\frac{2\lambda }{x^{2}}}{\left(1-e^{-\frac{\lambda }{x^{2}}}\right)}^{\theta_{1}-3}dx$

$-E\left(\frac{\partial^{2}\ln f_{CPF}\left(x;\theta_{1},\lambda \right)}{\partial \theta_{1}^{2}}\right)=\frac{1}{\theta_{1}^{2}}$

$-E_{\left(w_{i}\right)}\left(\frac{\partial^{2}\ln f_{CPF}\left(x;\theta_{1},\lambda \right)}{\partial \theta_{1}\partial \lambda }\right)=\frac{e^{-\frac{\lambda }{w_{i}^{2}}}}{w_{i}^{2}\left(1-e^{-\frac{\lambda }{w_{i}^{2}}}\right)}-\int_{w_{i}}^{\infty }\frac{2\theta_{1}\lambda x^{-5}e^{\frac{-\lambda }{x^{2}}}{\left(1-e^{\frac{-\lambda }{x^{2}}}\right)}^{\theta_{1}-2}}{{\left(1-e^{\frac{-\lambda }{w_{i}^{2}}}\right)}^{\theta_{1}}}dx$

$-E_{\left(w_{i}\right)}\left(\frac{\partial^{2}\ln f_{CPF}\left(x;\theta_{1},\lambda \right)}{\partial \lambda^{2}}\right)=\frac{1}{\lambda^{2}}+\theta_{1}\ln\left(1-e^{-\frac{\lambda }{w_{i}^{2}}}\right)-\left(\theta_{1}-1\right)\int_{w_{i}}^{\infty }\frac{2\theta_{1}\lambda x^{-7}e^{\frac{-\lambda }{x^{2}}}{\left(1-e^{\frac{-\lambda }{x^{2}}}\right)}^{\theta_{1}-3}}{{\left(1-e^{\frac{-\lambda }{w_{i}^{2}}}\right)}^{\theta_{1}}}dx$
